# Assessing the inequalities in demand for family planning satisfied in Ghana: evidence from the 1993–2014 Demographic and Health Surveys

**DOI:** 10.1093/inthealth/ihad019

**Published:** 2023-03-25

**Authors:** Simon Agongo Azure, Eugene Budu, Joshua Okyere, Felix Mensah, Bright Opoku Ahinkorah, Abdul-Aziz Seidu, Edward Kwabena Ameyaw

**Affiliations:** Population and Reproductive Health Division, School of Public Health, University of Port Harcourt, Choba, Nigeria; Department of Community Health, College of Health, Yamfo, Ghana; Korle Bu Teaching Hospital, P.O. Box 77, Accra, Ghana; Department of Population and Health, University of Cape Coast, Cape Coast, Ghana; Department of Nursing, College of Health Sciences, Kwame Nkrumah University of Science and Technology, Kumasi, Ghana; Department of Data Science and Economic Policy, University of Cape Coast, Cape Coast, Ghana; School of Public Health, Faculty of Health, University of Technology Sydney, Sydney, NSW, Australia; Centre for Gender and Advocacy, Takoradi Technical University, Takoradi, Ghana; College of Public Health, Medical and Veterinary Sciences, James Cook University, Townsville, QLD, Australia; School of Public Health, Faculty of Health, University of Technology Sydney, Sydney, NSW, Australia; L&E Research, Wa, Upper West Region, Ghana; Institute of Policy Studies and School of Graduate Studies, Lingnan University, Hong Kong, Hong Kong

**Keywords:** demand for family planning, Ghana, inequalities, satisfied

## Abstract

**Background:**

Family planning is essential in promoting the well-being of women and their families and communities and ensuring quality of care in contraceptive use. This study sought to assess the trend and inequalities in the demand for family planning satisfied (DFPS) in Ghana from 1993 to 2014.

**Methods:**

The World Health Organization's Health Equity Assessment Toolkit was employed to analyse the data. We disaggregated DFPS by five equity stratifiers: age, economic status, education, residence and region. Inequality was measured using difference (D), population attributable risk (PAR), ratio (R) and population attributable fraction (PAF).

**Results:**

The study showed an increment in DFPS from 17.8% to 38.7% between 1993 and 2014. With respect to age, we noted substantial inequality in 2003 (D=21.9 [95% confidence interval {CI} 15.2 to 28.7]), 1993 (D=4.8 [95% CI −1.8 to 11.4]) and 2014 (D=15 [95% CI 3 to 26.9]). The greatest economic inequality occurred in 1993 (PAF=69.7 [95% CI 50.8 to 88.6]; D=20.1 [95% CI 14.8 to 25.4]). Regarding education, significant inequality existed in 1993 (PAF=112 [95% CI 100.8 to 123.2]; D=29.7 [95% CI 21.9 to 37.4]). Ashanti and the Upper West regions reported significant inequalities (PAF=55.6 [95% CI 33.1 to 78.2]; D=16.1 [95% CI 8.9 to 23.3]).

**Conclusions:**

There are age-, education-, wealth-, residence- and region-related inequalities with respect to DFPS. Policymakers will have to prioritize the needs of women with no formal or low educational attainment in order to improve DFPS coverage. Special attention needs to be given to adolescent girls since they suffer greater inequalities than adult women.

## Introduction

Family planning is essential to promoting the well-being of women and their families and communities and ensuring quality of care in contraceptive use.^1^ Some of the common family planning methods include oral contraceptive pills, implants, injectables, patches, vaginal rings, intrauterine devices, condoms, male and female sterilization, lactation amenorrhea methods, withdrawal and fertility awareness methods.^2^ According to the World Health Organization (WHO),^2^ the number of women desiring to use family planning has increased over the past 2 decades, from 900 million in 2000 to nearly 1.1 billion in 2020. As a result of this increase, the number of women using a modern contraceptive method increased from 663 million to 851 million and the contraceptive prevalence rate increased from 47.7% to 49.0%. According to Ewerling et al.,^3^ in a study on the demand for family planning satisfied (DFPS), 52.9% of the women with a demand for family planning were using a modern contraceptive method, but coverage varied from one country to another. The results of the study further showed that West and Central Africa had the lowest coverage, with 32.9% using DFPS, whereas South Asia and Latin America and the Caribbean had the highest coverage, approximately 70% with DFPS.

In identifying factors associated with the difference in uptake of family planning, Ewerling et al.^3^ found that literacy, residence (urban or rural) and religion influenced the demand for modern contraceptives. In Kenya, a study on trends in inequalities in the use of family planning among women showed that the background characteristics of women, including age, marital status, education, wealth index, employment status, region and place of residence, have a significant influence on contraceptive use. Women who heard information on family planning through radio and television were more likely to use contraceptives compared with those who read newspapers or magazines.^4^

In sub-Saharan Africa, there are barriers associated with the unmet need for modern contraceptives. Some of these include fear of side effects, husband's disapproval, absence of menses and perception of the risk of pregnancy.^5^ The health implications of reducing these barriers and the unmet need for family planning has been shown to be positive. For instance, reduction of the unmet need for family planning averted approximately 56 million unintended pregnancies, 7 million unsafe abortions and 167 000 maternal deaths between 1993 and 2016.^6^

In Ghana, a study by Bawah et al.^7^ found that the prevalence of contraceptive use was low, at 13%, while unmet need is highly pervasive and demand for family planning is commonly for birth spacing rather than for the purpose of preventing pregnancy. Factors associated with this unmet need were similar to those in Ewerling et al.^3^ In addition, older women, being employed and women with a higher ideal number of children were identified as less likely to experience unmet need.^8^ Understanding the trend of inequalities in DFPS in Ghana from 1993 to 2014 using Demographic and Health Surveys (DHS) data will provide a picture of socio-economic differences in the country, allowing us to design interventions to increase uptake and conduct policy evaluation. The aim of this study was to assess the trend of inequalities in DFPS in Ghana from 1993 to 2014 using DHS data. Our study differs from the existing studies in that we focus on unearthing the trends in inequalities in DFPS, unlike previous studies that focused only on the associated factors of DFPS and the unmet need for family planning.^3,7,8^ Moreover, no study in Ghana has used the Health Equity Assessment Toolkit (HEAT) software to examine the inequalities across equity dimensions or assessed the variations in inequalities using complex summary measures.

## Methods

### Study design and data source

Data from the 1993, 1998, 2003, 2008 and 2014 Ghana DHS were analysed. The DHS data are collected in Ghana every 5 y. The 1988 data were exempted because that was the first survey year and there were inconsistencies with the other data points. In view of this, the study considered five data points (i.e. 1993, 1998, 2003, 2008 and 2014). The Ghana DHS forms part of the global surveys implemented by MEASURE DHS in about 85 low- and middle-income countries worldwide. The overarching focus of the DHS is to collate information on children, women and men. Among the cardinal issues captured are fertility and family planning. When sampling, selection of enumeration areas (EAs) is the first step and includes both rural and urban locations in Ghana. The complete sampling procedure has been elaborated in the final reports of the 1993, 1998, 2003, 2008 and 2014 Ghana DHS. The population for the study was all married and sexually active unmarried women aged 15–49 y. A total sample of 1820, 1737, 2101, 1693 and 3010 were drawn from the 1993, 1998, 2003, 2008 and 2014 Ghana DHS, respectively. Alternatively, we could have used the Multiple Indicator Cluster Surveys dataset; however, it has a lot of missing data and hence was not included in the study.

### Variables of interest

Study outcome was whether women had their DFPS or not. In the context of this study, DFPS is related to the indicator DFPS. Women who’s demand for family planning was satisfied were categorised as ‘1’, while those who did not have their demand for family planning satisfied were classified as ‘0’. Five stratifiers were used to assess inequality in demand for family planning satisfied: age (15–19 y, 20–49 y), economic status measured by wealth quintile (quintiles 1, 2, 3, 4, 5), education (no education, primary, secondary and above), place of residence (rural, urban) and region of residence (Western, Central, Greater Accra, Volta, Eastern, Ashanti, Brong Ahafo, Northern, Upper West, Upper East). In the DHS, the wealth quintile is a composite measure computed by combining data on a household's ownership of carefully identified assets, including a television, bicycle, materials used for house construction, sanitation facilities and type of water access. Principal component analysis was used to transform these variables into the wealth index by placing individual households on a continuous measure of relative wealth. The DHS segregates households into five wealth quintiles: poorest, poorer, middle, richer and richest. The quintiles were used in the final analysis. Education is measured by the highest level of formal education completed.^9^

### Statistical analysis

We used the 2019 updated HEAT version 3.1 software for all analyses.^10^ Five equity stratifiers were employed to disaggregate DFPS. These were age, economic status, education, residence and region of residence. Estimates and uncertainty intervals of DFPS with respect to the aforementioned stratifiers were computed. Four measures were used to compute inequality: difference (D), population attributable risk (PAR), population attributable fraction (PAF) and ratio (R). Two of these are simple unweighted measures (D and R) and two are complex weighted measures (PAR and PAF). At the same time, R and PAF are relative measures, whereas D and PAR are absolute measures. Summary measures were considered because the WHO has indicated that both absolute and relative summary measures are essential for generating policy-driven findings.^10^ Unlike simple measures, the complex ones take the size of categories inherent in a subpopulation into account. The WHO has extensively elaborated the procedure for generating summary measures.^10,11^

In calculating D in economic status, we subtracted women who had DFPS in the poorest group from women who had DFPS in the richest group. For education, we computed D as women who had DFPS with ‘no formal education’ minus women who had DFPS with ‘secondary/higher education’. The D for residence was computed by subtracting rural from urban residents. With respect to region, D was computed by subtracting the region with lowest estimate from the region with the highest estimate. We computed R for the variables with ordered responses such as education and wealth as the difference between the most-disadvantaged subgroup (lowest quintile and uneducated) and the most-advantaged subgroup (highest quintile and secondary or higher education). We derived PAR by computing the difference between women who had DFPS in the reference category (*y*_ref_) and the overall average of the prevalence of women who had DFPS. With respect to the ordered variables, *y*_ref_ referred to the most-advantaged subgroups. In the case of region, which was non-ordered, *y*_ref_ meant the region with the lowest estimate. The PAF was determined by distributing PAR by the overall average μ, multiplied by 100 (PAF=[PAR/μ]*100). A zero PAF or PAR means no inequality, while a higher value indicates a relatively greater inequality. Variation in women who had DFPS over the period was explored by referring to the 95% confidence intervals (CIs) of the survey years. Absence of overlap in the CI means that a statistically significant difference existed between the UIs, and vice versa. Sample weights were applied through the HEAT software to account for over- and undersampling.

### Trends in DFPS in Ghana, 1993–2014

The study showed an increment in DFPS from 17.8% to 38.7% between 1993 and 2014, as shown in Table 1. When considered across age categories, most women ages 20–49 y generally had a higher DFPS in 1993 (18.1%) and this was sustained through 2014 (39%). DFPS was highest among the richest women in the 1993 survey (30.2%), however, those in the second wealth quintile dominated in 2014 (41.3%). In 1993, the analysis indicated that a significant proportion of women who had attained secondary education or higher reported DFPS (37.7%), while at least 4 of 10 women with primary education had DFPS in 2014 (44.0%). A significant proportion of urban residents indicated DFPS in 1993 (24.7%), however, DFPS was greater among rural residents in 2014 (41.8%). Across the regions of residence, DFPS was greater among Greater Accra women (27.7%) in 1993, with women from the Upper West region leading in 2014 (46.8%), as shown in Table 1.

**Table 1.** Trends in DFPS in Ghana, 1993–2014

	1993 (17.8%)		1998 (24%)		2003 (31.5%)		2008 (28.2%)		2014 (38.7%)	
Dimension	n	% (95% CI)	n	% (95% CI)	n	% (95% CI)	n	% (95% CI)	n	% (95% CI)
Age (years)										
15–19	98	13.3 (8.1 to 21.1)	84	18.3 (11.5 to 27.8)	90	10.5 (5.6 to 18.8)	64	10.1 (4.6 to 20.8)	72	24.1 (14.4 to 37.5)
20–49	1722	18.1 (16.3 to 19.9)	1653	24.3 (22 to 26.6)	2011	32.5 (30.2 to 34.9)	1629	28.9 (26.3 to 31.6)	2938	39 (36.3 to 41.9)
Economic status										
Quintile 1 (poorest)	287	10.1 (7 to 14.4)	358	15.8 (12.4 to 20)	412	15.8 (12.3 to 20)	289	22.9 (17.9 to 29)	541	39.8 (35.5 to 44.4)
Quintile 2	326	11 (8.1 to 14.9)	302	20.9 (16.4 to 26.2)	424	31 (25.7 to 36.9)	364	22.8 (18.1 to 28.1)	574	41.3 (35.9 to 46.8)
Quintile 3	386	14 (10.8 to 18)	354	22 (17.3 to 27.5)	412	31.2 (26 to 37)	322	25.8 (21.1 to 31.2)	589	40.9 (35.8 to 46.2)
Quintile 4	394	19.3 (15.4 to 23.9)	340	29.2 (23.7 to 35.4)	431	34.3 (28.5 to 40.5)	384	31.3 (26.2 to 37)	641	37 (32.5 to 41.8)
Quintile 5 (richest)	427	30.2 (26.6 to 34.1)	382	31.3 (26.3 to 36.7)	421	45 (40.3 to 49.7)	334	37.2 (31.1 to 43.7)	667	35.1 (29.3 to 41.5)
Education										
No education	607	8.1 (6.1 to 10.6)	523	18.8 (15.3 to 22.9)	682	21.9 (18.8 to 25.3)	415	22.2 (18.4 to 26.6)	707	36 (32.2 to 40)
Primary school	1054	20.4 (18.1 to 22.9)	340	21.9 (17.3 to 27.3)	466	31.6 (26.7 to 36.8)	432	26.6 (22.3 to 31.3)	594	44 (38.8 to 49.4)
Secondary school or higher	159	37.7 (30.6 to 45.4)	874	27.9 (24.5 to 31.5)	952	38.4 (35.2 to 41.8)	847	31.9 (28.3 to 35.7)	1709	37.9 (34.5 to 41.5)
Place of residence										
Rural	1163	13.9 (11.9 to 16.2)	1161	21.2 (18.6 to 24.1)	1248	25.2 (22.4 to 28.3)	970	25.9 (22.6 to 29.4)	1559	41.8 (38 to 45.7)
Urban	657	24.7 (22 to 27.6)	575	29.6 (25.8 to 33.7)	853	40.7 (37.3 to 44.3)	723	31.2 (27.5 to 35.2)	1452	35.3 (31.6 to 39.2)
Region										
Ashanti	327	13.5 (10.7 to16.8)	276	24.9 (18.9 to 32.1)	3745	36 (29.7 to 42.7)	344	24.7 (19.8 to 30.4)	564	34.7 (27 to 43.2)
Brong Ahafo	203	21.2 (15.7 to 28)	143	24.2 (17 to 33.3)	256	38.5 (34 to 43.2)	172	33.6 (26.4. to 41.6)	249	46.3 (38.5 to 54.3)
Central	175	13.1 (10 to 17.1)	191	23.2 (17.4 to 30.3)	178	20.3 (13.6 to 29.3)	185	23.4 (16 to 32.9)	321	45.4 (39.9 to 51.1)
Eastern	217	20.3 (16.2 to 25)	253	33 (26.7 to 39.9)	216	35.3 (28.1 to 43.2)	161	26.6 (19 to 35.9)	323	39.1 (33.1 to 45.5)
Greater Accra	231	27.7 (22.5 to 33.6)	271	28.9 (24.2 to 34.1)	310	39.8 (33.4 to 46.7)	250	37.6 (30.3 to 45.5)	574	32.8 (26 to 40.4)
Northern	152	12.5 (8 to 19)	82	13.5 (8 to 21.9)	198	16.8 (12.9 to 21.5)	128	15 (8.9 to 24.3)	219	27.8 (23 to 33.2)
Upper East	89	19.1 (9.4 to 34.9)	39	22.4 (13.7 to 34.5)	121	19 (10.8 to 31.2)	79	30.6 (21.4 to 41.6)	110	46.5 (41.2 to 51.9)
Upper West	48	14.6 (5.6 to 33)	77	20.3 (14.1 to 28.3)	58	38.3 (32.2 to 44.8)	41	41.2 (34.8 to 47.8)	77	46.8 (39.3 to 54.5)
Volta	232	11.6 (7.8 to 17.1)	203	19.9 (14 to 27.7)	195	30 (22.4 to 38.9)	183	32.6 (25.3 to 40.9)	278	42.7 (34.9 to 50.9)
Western	146	24.7 (18.9 to 31.5)	201	15.3 (10.4 to 22.1)	194	29 (24.8 to 33.6)	153	22.6 (17.2 to 29.1)	298	42.8 (35.3 to 50.7)

### Inequality indices of estimates of factors associated with DFPS: use of modern and traditional methods, 1993–2014

Table 2 shows the indices of estimates of factors associated with DFPS. We observed inequality in factors associated with DFPS as revealed by the simple (D and R) and complex (PAF and PAR) measures. With respect to age, we noted substantial inequality in 2003 as revealed by the simple measure (D=21.9 [95% CI 15.2 to 28.7]) as compared with 1993 (D=4.8 [95% CI −1.8 to 11.4]) and 2014 (D=15 [95% CI 3 to 26.9]). Analysis of economic status showed that the greatest inequality occurred in 1993 (PAF=69.7 [95% CI 50.8 to 88.6]; D=20.1 [95% CI 14.8 to 25.4]). As of 2014, there seemed to be no inequality in DFPS except in one of the simple measures, which showed a slight inequality (R=0.9 [95% CI 0.7 to 1.1]). Regarding education, significant inequality existed in 1993, as shown by both the complex (PAF 112 [95% CI 100.8 to 123.2]) and simple (D 29.7 [95% CI 21.9 to 37.4]) measures. The education-based inequality decreased substantially in 2014 (R=1.1 [95% CI 0.9 to 1.2]). The residence-based inequality decreased immensely between 1993 and 2014. In 1993, the analysis revealed significant inequality as shown by the complex (PAF=38.5 [95% CI 30.8 to 46.2]) and simple (D=10.7 [95% CI 7.2 to 14.2]) indicators. In the case of 2014, the variation was shown by the complex measures. For region, both simple (D=16.1 [95% CI 8.9 to 23.3]) and complex (PAF 55.6 [95% CI 33.1 to 78.2]) indicators revealed significant inequality between the Ashanti and the Upper West regions. This persisted until 2014 (D 19.1 [95% CI 9.9 to 28.2], PAF=21.1 [95% CI 6.1 to 36.1]), as shown in Table 2.

**Table 2.** Inequality indices of estimates of factors associated with DFPS, 1993–2014

	1993			1998			2003			2008			2014		
Dimension	Est.	LB	UB	Est.	LB	UB	Est.	LB	UB	Est.	LB	UB	Est.	LB	UB
Age															
D	4.8	−1.8	11.4	6	−2.4	14.4	21.9	15.2	28.7	18.7	10.6	26.9	15	3	26.9
PAF	1.5	−37	39.9	1.2	−33.7	36.1	3	−18.2	24.2	2.5	−25.8	30.9	0.9	−25.1	26.9
PAR	0.3	−6.6	7.1	0.3	−8.1	8.7	0.9	−5.8	7.6	0.7	−7.3	8.7	0.4	−9.7	10.4
R	1.4	0.8	2.2	1.3	0.8	2.1	3.1	1.7	5.7	2.8	1.3	6.1	1.6	1	2.6
Economic status															
D	20.1	14.8	25.4	15.5	9.1	21.9	29.2	23.2	35.3	14.2	5.8	22.6	−4.7	−12.3	2.9
PAF	69.7	50.8	88.6	30.5	15.8	45.1	42.7	32.1	53.2	32	16	47.9	0	−9.7	9.7
PAR	12.4	9	15.8	7.3	3.8	10.8	13.5	10.1	16.8	9	4.5	13.5	0	−3.7	3.7
R	3	2	4.4	2	1.5	2.6	2.9	2.2	3.7	1.6	1.2	2.2	0.9	0.7	1.1
Education															
D	29.7	21.9	37.4	9	3.9	14.2	16.6	11.9	21.2	9.6	4.1	15.1	1.9	−3.3	7.2
PAF	112	100.8	123.2	16.2	4	28.3	21.9	13.4	30.4	13.2	0.5	25.9	0	−8	8
PAR	19.9	17.9	21.9	3.9	1	6.8	6.9	4.2	9.6	3.7	0.1	7.3	0	−3.1	3.1
R	4.7	3.3	6.6	1.5	1.2	1.9	1.8	1.5	2.1	1.4	1.2	1.8	1.1	0.9	1.2
Place of residence															
D	10.7	7.2	14.2	8.4	3.6	13.1	15.5	11	20	5.4	0.2	10.5	−6.5	−11.8	−1.1
PAF	38.5	30.8	46.2	23.3	17.2	29.4	29.2	23.9	34.5	10.9	4.3	17.5	0	−4.3	4.3
PAR	6.9	5.5	8.2	5.6	4.1	7.1	9.2	7.5	10.9	3.1	1.2	4.9	0	−1.7	1.7
R	1.8	1.5	2.1	1.4	1.2	1.7	1.6	1.4	1.9	1.2	1	1.4	0.8	0.7	1
Region															
D	16.1	8.9	23.3	19.5	10	28.9	23	15.2	30.9	26.1	16.2	36.1	19.1	9.9	28.2
PAF	55.6	33.1	78.2	37.6	5.6	69.7	26.3	10.1	42.6	46.2	24.2	68.3	21.1	6.1	36.1
PAR	9.9	5.9	13.9	9	1.3	16.7	8.3	3.2	13.4	13	6.8	19.2	8.2	2.4	14
R	2.4	1.5	3.7	2.4	1.4	4.2	2.4	1.8	3.2	2.7	1.6	4.6	1.7	1.3	2.2

Est: estimate; LB: lower boundary; UB: upper boundary.

## Discussion

The present study sought to assess inequalities in DFPS over time. Our findings show a general increase in DFPS from 17.8% in 1993 to 38.7% in 2014. The observed current DFPS coverage (38.7%) is higher than the <20% coverage that has been reported in other African countries, including Chad, Democratic Republic of Congo and Benin.^3^ We found that across the surveys and within the respective survey years, women with secondary or higher education had higher DFPS as compared with their counterparts with no education or only primary education. This finding is corroborated by an earlier study that found DFPS to be high among women with secondary or higher education.^12^ The result is consistent with the findings of a related study conducted in Ghana.^13^ Higher formal education tends to increase women's autonomy, as well as enhance their decision-making capacity with respect to accessing or using modern contraceptives.^14,15^ This may explain the consistently high DFPS among women with secondary or higher education as opposed to those with no formal education or only primary education. Relatedly, the study showed that there was substantial inequality in DFPS coverage in 1993; however, this inequality decreased significantly in 2014. Perhaps this finding may be explained by the significant increase in the number of adolescent girls and women who have received formal education over time.

Consistently across the various survey points as well as within each survey year, it was revealed that adults (i.e. 20–49 y of age) had higher DFPS compared with adolescents (i.e. 15–19 y of age). We also found substantial inequality in 2003 as compared with 1993 and 2014. This result is analogous to findings reported in the Tigray region of Ethiopia, with high use of modern contraceptives among women >20 y of age.^16^ The age-related inequalities as illuminated by our findings are unsurprising, as several studies have shown that adolescent girls face innumerable barriers in their quest to access and use modern contraceptives and family planning methods.^13,17^

Our study also found wealth-related inequalities with respect to DFPS among Ghanaian women. Within the respective survey years, DFPS coverage was low among women from the poorest wealth quintile, except for 2014, where those in the second wealth quintile dominated, thus suggesting a reduction in wealth-related inequalities in the context of DFPS over time. Similar findings have been reported in related studies conducted in Ghana^13^ and sub-Saharan Africa.^18^ Previous studies have shown that women in the richest wealth quintile often prefer and use long-term modern contraceptive methods as compared with those in lower wealth quintiles.^13,19^ These long-term methods that are preferred by women in the richest wealth quintile tend to be expensive, thus limiting DFPS. This may be a reason for the low DFPS among women in the richest wealth quintile compared with women in the second wealth quintile in the 2014 survey. Our study also revealed that the greatest inequalities in DFPS were in 1993, while in 2014 there seemed to be no wealth-related inequalities. This change in wealth-related inequalities within the context of DFPS could be due to the increase in health promotion programs that target women of disadvantaged economic status.

In agreement with an earlier study conducted in Ghana,^13^ our study found a significant decrease in residence-related inequalities in DFPS over time. The most significant inequality was reported in 1993 and the lowest inequality was reported in 2014. This is an indication of how Ghana as a country has fared in reducing rural–urban inequalities with respect to DFPS. Between 1993 and 2014, several interventions and policies have been implemented to enhance DFPS. Notable among these is the Community-based Health Planning and Services (CHPS) initiative, which was established to narrow the rural–urban gap in accessing healthcare services and improve universal health coverage.^20,21^ This initiative, among others, brings rural women closer to healthcare facilities where they can easily access modern contraceptives and family planning methods, thus contributing to the reduction in residence-related inequalities in terms of DFPS.

Finally, our study revealed region-related inequalities with respect to DFPS. Across the regions of residence of the surveyed women, DFPS was high among Greater Accra women in 1993, but the Upper West region reported the highest DFPS in 2014. Additionally, both the simple and complex inequality indicators revealed significant inequality between the Ashanti and Upper West regions that persisted until 2014. The reasons for the observed region-related inequalities are unclear; however, this might be an effect of existing education- and income-related inequalities across the various regions.^22^

### Policy implications

Our finding concerning the education-related inequalities in DFPS coverage emphasizes the needs of women with no formal or low educational attainment and the need to develop policies and interventions that are tailored to these women with respect to modern contraceptives and family planning. The observed age-related inequalities in DFPS coverage highlight the need for the incorporation of pro-adolescent components into policies, programs and interventions designed to advance DFPS.

### Strengths and limitations

The strength of our study lies in the robustness of our data analyses. We used both simple and complex measures of inequality. Also, the use of the Ghana DHS, which is a nationally representative dataset, ensured that our findings are generalizable to the population of Ghanaian women in the reproductive age group. However, there are limitations that need to be considered when interpreting the study findings. Our use of secondary data limits the selection the variables. Also, residual variables such as ethnicity, religion and health insurance coverage were not included in the present study, thus limiting our findings.

## Conclusions

The present study reveals the existence of age-, education-, wealth-, residence- and region-related inequalities with respect to DFPS. We found a general increase in DFPS over time. However, policymakers will have to prioritize the needs of women with no formal or low educational attainment in order to improve DFPS coverage. Additionally, special attention needs to be given to adolescent girls, as they suffer greater inequalities than adult women with regards to DFPS. Future studies could adopt a qualitative method to explore the reasons behind the continued decrease in DFPS in the Greater Accra region over the last three Ghana DHS.

## Data Availability

The dataset is freely available for download at https://dhsprogram.com/data/available-datasets.cfm.
